# Editorial: Novel therapies and unexpected challenges: overcoming immune-mediated toxicities in modern oncology

**DOI:** 10.3389/fmed.2026.1810214

**Published:** 2026-03-13

**Authors:** Igor Age Kos, Salvatore Fiorenza, Michel Michels de Oliveira, Dominic Kaddu-Mulindwa

**Affiliations:** 1 Department of Internal Medicine I, University of Saarland, Homburg, Germany; 2 Faculty of Medicine and Health, University of Sydney, Sydney, NSW, Australia; 3 Department of Haematology, Alfred Health, Melbourne, VIC, Australia; 4 Epworth Centre for Immunotherapies and Snowdome Laboratories, Melbourne, VIC, Australia; 5 CIONC, Centro Integrado de Oncologia de Curitiba, Curitiba, Brazil; 6 Centre Hospitalier du Nord, Ettelbruck, Luxembourg

**Keywords:** bispecific antibodies (BsAbs), cancer, CAR T, checkpoint inhibition (CPI), cytokine release syndrome (CRS), hematology, lymphoma, safety

While novel cellular and immune-based therapies have fundamentally reshaped oncology, they have also introduced a complex new spectrum of immune-mediated adverse events (1, 2). Management of these toxicities frequently relies on corticosteroid-based regimens, which can be effective and lead to symptom control (1, 2). However, this strategy has important drawbacks: steroids may reduce the anti-tumor efficacy of immune-based therapies, and steroid-induced immunosuppression increases the risk of infection and may adversely affect non-relapse morbidity and mortality (3). These challenges call for rigorous post-marketing surveillance of advanced therapeutic medicinal products and focused clinical and translational efforts to describe rare and unexpected adverse events and develop tools for the early recognition, risk stratification, and optimal management of recurrent and clinically significant toxicities.

In the Frontiers Research Topic “*Overcoming side effects in patients undergoing immunotherapies and cell therapies: a deeper evaluation of advanced therapeutic medicinal products*,” these issues are addressed from complementary perspectives.

Immune checkpoint inhibitors (ICIs) have improved survival rates across a broad spectrum of malignancies and are currently the standard of care in multiple therapeutic settings. At the same time, they have introduced new immune-mediated toxicities (4). Cutaneous adverse events are among the most frequent and are usually manageable, but in rare cases, severe, life-threatening reactions such as Stevens–Johnson syndrome (SJS) and toxic epidermal necrolysis (TEN) can occur (2). In their systematic review, Zhou et al. described 50 patients from 47 publications. The authors reported a 20% mortality rate, with infections and tumor progression being the leading causes of death, likely reflecting treatment-related immunosuppression. Events typically occurred early (with a median of 23 days after ICI initiation), and death followed after a median of 28 days after symptom onset. A poor outcome was associated with a TEN phenotype and larger body surface area involvement, while dual ICI therapy correlated with a higher rate of TEN than monotherapy (Zhou et al.). Corticosteroids and intravenous immunoglobulins were the most frequently used treatments, but in this small cohort, no regimen showed clear superiority. These findings underscore the need for vigilant early monitoring and better therapeutic strategies for suspected ICI-related SJS/TEN (Zhou et al.).

Bispecific antibodies (BsAbs) have rapidly expanded the therapeutic options for relapsed or refractory B-cell lymphomas by offering potent T-cell-redirecting activity (5, 6). As their use becomes routine, a systematic understanding of their toxicity spectrum has become essential to optimizing outcomes. A review by Doig and Yannakou provided a comprehensive synthesis of the acute and delayed adverse events (AEs) associated with currently approved BsAbs. Cytokine release syndrome (CRS) remains the most common AE, typically manifesting early and predominantly in low grade; however, it requires vigilant, risk-adapted mitigation strategies. The manuscript underscores how step-up dosing, premedication, and standardized intervention algorithms have improved the safety without compromising efficacy (Doig and Yannakou). Neurotoxicity, including immune-effector cell-associated neurotoxicity syndrome (ICANS), although less frequent than with CAR T cell therapy, still occurs (Doig and Yannakou). The authors reinforced the importance of early recognition and of differentiation from disease-related symptoms or other non-immune complications. The review expands on infection risks, cytopenias, and prolonged immunosuppression, which are particularly relevant in heavily pre-treated populations. The authors explain how B-cell depletion, T-cell exhaustion, and sustained cytokine perturbations may contribute to long-term immune dysfunction and infection risk.

As clinical experience with treatment-emergent adverse events such as CRS increases, new therapeutic approaches are being implemented. For example, IL-6 receptor blockade with tocilizumab is recommended for CRS after CAR T cell therapy and other T-cell–engaging treatments. Unlike corticosteroids, tocilizumab does not appear to compromise anti-tumor efficacy or long-term outcomes (1). Further advances require understanding the mechanisms driving toxicity. In their article, Mazein et al. presented an integrative conceptual model of CRS as an adverse outcome. By combining immunotoxicology and systems biology approaches, they constructed an immune-related Adverse Outcome Pathways (irAOP)-based CRS Map that harmonized immune-related AOPs across five CRS-inducing modalities: CAR T cells, checkpoint inhibitors, T cell-engaging bispecifics, monoclonal antibodies targeting T cell receptors, and FcγR-activating antibodies into a mechanistic network. The CRS Map, published on the MINERVA platform, encompasses 24 cell types, 425 entities, and 430 interactions and can be interactively explored and used for data visualization and network analyses, including the overlay of clinical cytokine datasets (Mazein et al.). This explorable resource supports hypothesis generation, biomarker discovery, and future *in silico* modeling that may inform CRS management, prediction, and prevention.

Persistent cytopenias following CAR T-cell therapy pose a challenge to clinicians and patients, as it becomes especially important to manage their consequences: infection, bleeding, and anemia. Predictive tools can help identify patients at higher risk of developing cytopenias. The CAR-HEMATOTOX is a simple bedside score composed of biomarkers representing a poor hematopoietic reserve (hemoglobin, platelet, and neutrophil counts) and inflammatory stressors on marrow function (ferritin and C-reactive protein, CRP) (7). This score has been shown to predict the depth and length of neutropenia, infectious complications (8), and even progression-free survival (PFS) and overall survival (OS) (9). In this Research Topic, Lesan et al. further validated this score in an independent German cohort across multiple CAR T products. Despite the small size of their study, the authors identified key predictors of cytopenias following CAR T cell therapy. In particular, early thrombocytopenia and neutropenia at the time of CRS were found to be strongly associated with CAR-HEMATOTOX, especially pre-existing thrombocytopenia (Lesan et al.). This is important as this is the timepoint when patients receive immunosuppression for CRS but would equally be at risk of developing infections. In a following phase, high CAR-HEMATOTOX was found to be associated with anemia. Although this study did not confirm an association between CAR-HEMATOTOX and PFS or OS, the findings support the use of the CAR-HEMATOTOX score prior to infusion and referencing it in post-infusion clinical decision-making.

Taken together, this Research Topic provides essential insights into immune-related adverse events across multiple domains, including real-world evidence, integrative systems approaches, and systematic literature reviews. As treatments become more complex, pharmacovigilance and high-quality reporting remain crucial to improve our understanding of the safety profiles of novel mechanisms of action, with the potential to ultimately influence patient outcomes. In parallel, a deeper understanding of the underlying mechanisms of these toxicities can inform clinical decision-making and support more personalized and effective management strategies.
